# The Immunophenotype and the Odontogenic Commitment of Dental Pulp Stem Cells Co-Cultured with Macrophages Under Inflammatory Conditions Is Modulated by Complex Magnetic Fields

**DOI:** 10.3390/ijms26010048

**Published:** 2024-12-24

**Authors:** Marialucia Gallorini, Noemi Mencarelli, Natalia Di Pietro, Viviana di Giacomo, Susi Zara, Alessia Ricci, Monica Rapino, Adriano Piattelli, Alessandro Cipollina, Amelia Cataldi

**Affiliations:** 1 Department of Pharmacy, “G. d’Annunzio” University of Chieti-Pescara, Via dei Vestini 31, 66100 Chieti, Italy; marialucia.gallorini@unich.it (M.G.); noemi.mencarelli@phd.unich.it (N.M.); viviana.digiacomo@unich.it (V.d.G.); susi.zara@unich.it (S.Z.); alessia.ricci@unich.it (A.R.); 2 Department of Medical, Oral and Biotechnological Sciences, “G. d’Annunzio” University of Chieti-Pescara, Via dei Vestini 31, 66100 Chieti, Italy; natalia.dipietro@unich.it; 3 Genetic Molecular Institute of CNR, Unit of Chieti, “G. d’Annunzio” University of Chieti-Pescara, 66100 Chieti, Italy; m.rapino@unich.it; 4 School of Dentistry, Saint Camillus International University Rome, Via di Sant’Alessandro, 00131 Rome, Italy; apiattelli51@gmail.com; 5 Independent Researcher, 92019 Sciacca, Italy; alexandros1960@libero.it; 6 UdA Tech Lab, “G. d’Annunzio” University of Chieti-Pescara, Via dei Vestini 31, 66100 Chieti, Italy

**Keywords:** dental pulp stem cells, macrophages, complex magnetic fields, inflammation, regenerative medicine

## Abstract

Dental inflammatory diseases remain a challenging clinical issue, whose causes and development are still not fully understood. During dental caries, bacteria penetrate the tooth pulp, causing pulpitis. To prevent pulp necrosis, it is crucial to promote tissue repair by recruiting immune cells, such as macrophages, able to secrete signal molecules for the pulp microenvironment and thus to recruit dental pulp stem cells (DPSCs) in the damaged site. To date, root canal therapy is the standard for dental caries, but alternative regenerative treatments are gaining attention. Complex Multifrequency Magnetoelectric Fields (CMFs) represent an interesting tool due to their potential anti-inflammatory activity. Against this background, the present work aims at investigating whether the CMF treatment might restore redox balance in a co-culture model of DPSCs and inflamed macrophages mimicking an inflammatory condition, like pulpitis. Results show that superoxide anion levels and markers related to the polarization of macrophages are modulated by the CMF treatment. In parallel, the use of CMFs discloses an impact on the odontogenic commitment of DPSCs, their immunophenotype being considerably modified. In conclusion, CMFs, by modulating the odontogenic commitment and the anti-inflammatory response of DPSCs, might represent a suitable therapeutic tool against pulpitis and, in general, towards dental inflammatory diseases.

## 1. Introduction

Although with increased awareness and attention on oral health, inflammatory dental diseases remain a challenging clinical issue, whose causes and development are still not fully understood. Dental caries, a common and chronic degenerative condition, are caused by bacterial infections and can disclose detrimental outcomes if untreated [1].

During dental caries, bacteria can erode the dentin layer and penetrate the tooth pulp through dentinal tubules or deep cavities, leading to pulpitis and periapical inflammation, which can eventually result in tooth loss [2]. In pulpitis [3], the significant activation and infiltration of inflammatory cells, such as T-lymphocytes, monocytes, macrophages, and eosinophils, occurs in response to bacterial products, such as lipopolysaccharide (LPS). To prevent pulp necrosis at the early stages of pulpitis, it is therefore crucial to promote tissue repair [4].

The immune response in the injured pulp compartment fosters a favorable environment for tissue regeneration because immunocompetent cells can secrete signal molecules and thus recruit dental pulp stem cells (DPSCs) to the damaged site. DPSCs subsequently proliferate and migrate into the injured area, differentiate into odontoblast-like cells, and form reparative dentine [5,6]. However, a key challenge concerning stem cells is maintaining their stemness phenotype. Cell redox equilibrium and reactive oxygen species (ROS)-related signaling appear to play a crucial role in balancing self-renewal and differentiation [5].

Currently, the primary treatment of pulpitis is represented by the root canal therapy. However, controlling intracanal and pulp infections due to a degeneration of dental caries and during dental practice is challenging, and many disinfectants used in root canal treatments often can even cause irritation to the surrounding peri-radicular tissues [7]. In this scenario, research is increasingly focused on developing innovative therapeutic anti-inflammatory strategies for the regeneration of pulp tissue. In the field of non-invasive therapies, Complex Magnetic Fields (CMFs) of low and very low frequency and intensity represent an interesting tool due to their potential anti-inflammatory and antioxidant activities. It has been therefore reported that CMFs are able to reduce ROS production and the expression of important pro-inflammatory mediators for macrophage polarization, such as TNFα, IL-6, and iNOS [8,9]. However, intracellular molecular processes underlying these beneficial effects are still not fully understood, and more in-depth studies are necessary to increase knowledge about the biological and molecular mechanisms triggered by CMFs.

Against this background, the present work aims at investigating whether the CMF treatment might restore the redox balance in a co-culture model of DPSCs and LPS-stimulated macrophages mimicking an inflammatory condition, like pulpitis. To this aim, the generation of superoxide anion levels and the modulation of markers related to the polarization of macrophages were measured. Moreover, given that mesenchymal stem cells in the DPSC population can self-renew and differentiate into multiple lineages, the potential proliferative effect of the CMF treatment and its impact on cell stemness were also examined by monitoring the cell surface markers related to the odontogenic commitment.

## 2. Results

### 2.1. Morphological Changes After the CMF Treatment

**Figure 1:**
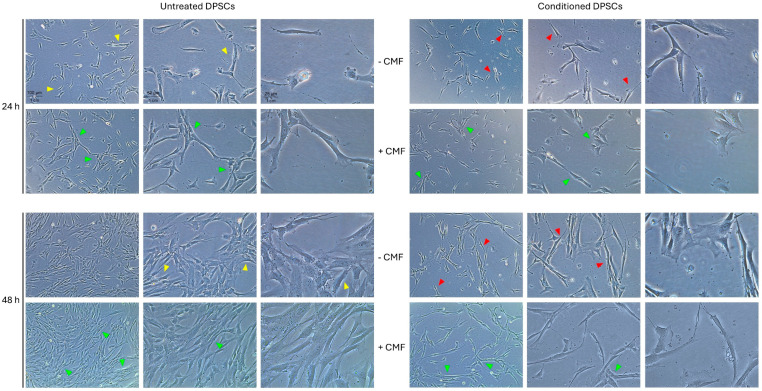
Light phase-contrast images of DPSCs exposed to the CMF treatment under basal and pro-inflammatory conditions after 24 and 48 h. Magnification 10×, 20×, and 40×. Arrows highlight morphological changes towards treatments. Yellow arrows: basal conditions; red arrows: effects of conditioned medium; green arrows: effects of CMF.

**Figure 2:**
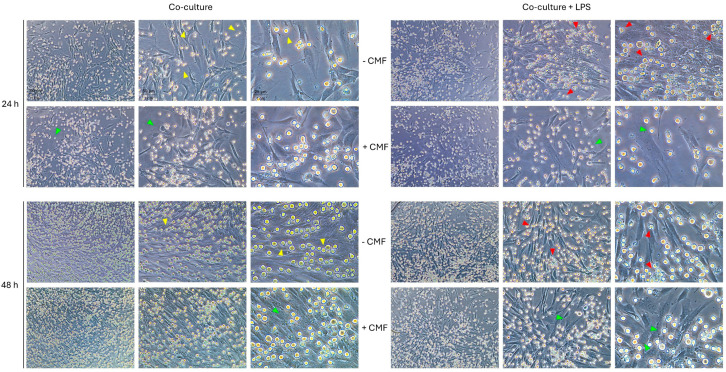
Light phase-contrast images of the DPSCs/macrophages co-culture model exposed to the CMF treatment under basal and pro-inflammatory conditions after 24 and 48 h. Magnification 10×, 20×, and 40×. Arrows highlight morphological changes towards treatments. Yellow arrows: basal conditions; red arrows: effects of LPS; green arrows: effects of CMF.

The effect of CMF treatment was evaluated on untreated and conditioned DPSCs and on the co-culture model (Figure 1 and Figure 2).

Exposure to the medium from LPS-treated macrophages significantly affected DPSCs behavior and morphology (Figure 1, red arrows). DPSCs, which typically exhibit a spindle-shaped morphology with elongated bodies and tapering ends (Figure 1, yellow arrows), undergo a morphological change, becoming thinner and more elongated, with a reduction in size. Moreover, cell proliferation dramatically decreases, mainly after 48 h. CMF treatment markedly enhances the proliferation of untreated cells. While a slight increase in the proliferation of conditioned cells is observed, more notably, a restoration of the spindle-shaped morphology is detected after 48 h (Figure 1, green arrows).

The pro-inflammatory stimulus induces macrophage proliferation increase and clustering (Figure 2, red arrows). Additionally, they exhibit a less smooth surface, and a more irregular shape compared to untreated macrophages. No alterations are observed in DPSC morphology, but a slight reduction in their proliferation is detected. The main effect of CMF treatment is a reduction in the presence of macrophages, especially within the first 24 h (Figure 2, green arrows).

### 2.2. Cytotoxicity Occurrence Under Pro-Inflammatory Conditions and After CMF Treatment

**Figure 3:**
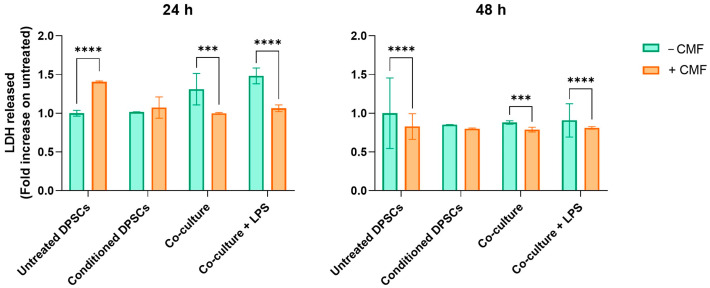
LDH released from cells exposed to the CMF treatment under basal and pro-inflammatory conditions after 24 and 48 h. The bar graphs show the LDH released as the fold increase on the untreated control (set as 1). *** *p* < 0.001 and **** *p* < 0.0001.

The LDH release was measured to evaluate cell membrane integrity and overall cell health under different experimental conditions and upon CMF treatment. A moderate increase in LDH release is detected in untreated co-cultures within the first 24 h (Figure 3). At 48 h, no differences are observed between the untreated experimental conditions. CMF treatment consistently reduces LDH release in the co-culture model, both in LPS-stimulated and unstimulated cells and after 24 and 48 h of treatment.

### 2.3. The Effect of CMF Treatment on the Generation of Superoxide Anions

**Figure 4:**
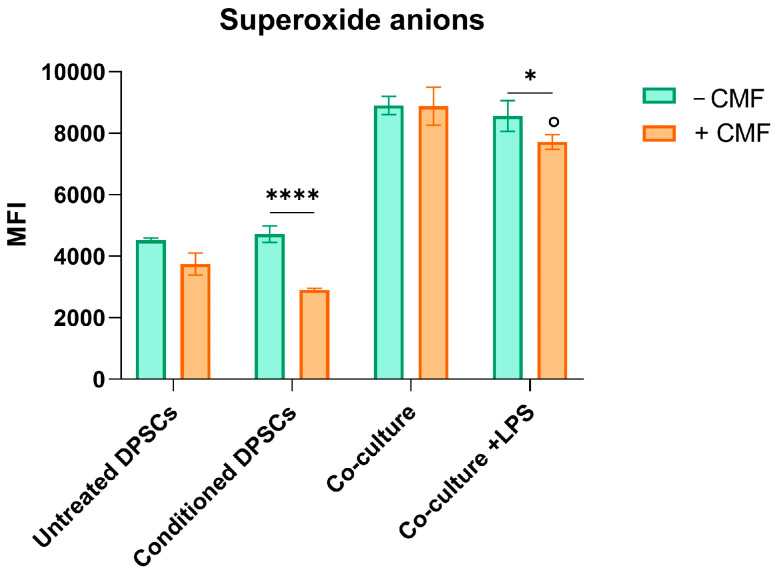
Generation of superoxide anions in cells exposed to CMF treatment after 24 h. Bar graph represents intracellular superoxide anions proportional to MFI (mean fluorescence intensity) in the phycoerythrin channel (FL-2). * *p* < 0.01 and **** *p* < 0.0001; ˚ *p* < 0.05 between co-cultured cells and co-cultured cells with LPS in the presence of CMF.

Superoxide anion levels were measured to investigate the oxidative state of the cells at 24 h, considering the role of Reactive Oxygen Species (ROS) in various signaling pathways. Superoxide anion levels increase two-fold when DPSCs are co-cultured with macrophages. CMF treatment reduces superoxide anion generation in all experimental conditions, except in the co-culture, where no significant changes are observed. In conditioned DPSCs, superoxide anion levels decrease by 50% upon CMF exposure (Figure 4).

### 2.4. Immunophenotypic Profile of Co-Cultured DPSCs and Macrophages Under Inflammatory Conditions and After CMF Treatment

**Figure 5:**
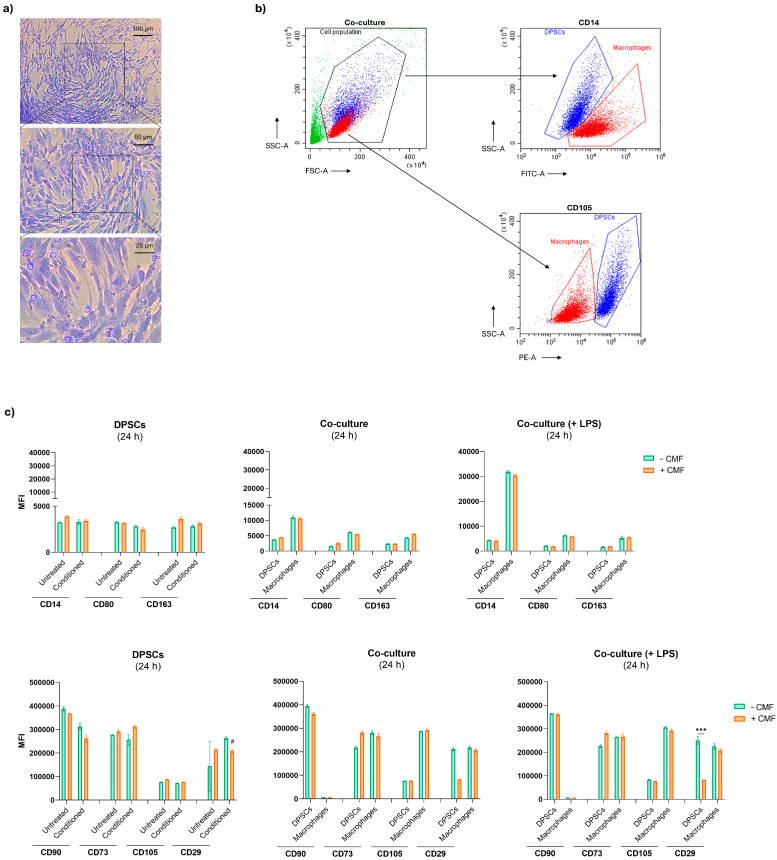
Immunophenotypic profile of cells exposed to the CMF treatment after 24 h. (**a**) DPSCs and macrophages co-cultured under basal conditions stained with crystal violet. Magnification 10×, 20×, and 40×. (**b**) Gating strategy for the immunophenotype analysis performed by flow cytometry. The Side scatter/Forward scatter (SSC/FSC) dot plot represents morphological parameters of co-cultured DPSCs (in blue) and macrophages (in red). Side scatter/Fluorescein (SSC/FITC) dot plot represents cells stained positive for CD14-FITC and was used to discriminate DPSCs (stained negative) and macrophages (stained positive). Side scatter/Phychoerythrin (SSC/PE) dot plots represent cells stained positive for CD105-PE and were used to discriminate DPSCs (stained positive) and macrophages (weakly stained positive). (**c**) Immunophenotypic profile of cells in different experimental conditions. The positivity of cells for CD14-FITC, CD80-PE, CD163-PE, CD90-FITC, CD73-PE, CD105-PE, and CD29-PE is proportional to the MFI (mean fluorescence intensity). *** *p* < 0.0001 between cells exposed to the CMF treatment and cells not exposed; # *p* < 0.01 between conditioned DPSCs and untreated DPSCs without CMF.

**Figure 6:**
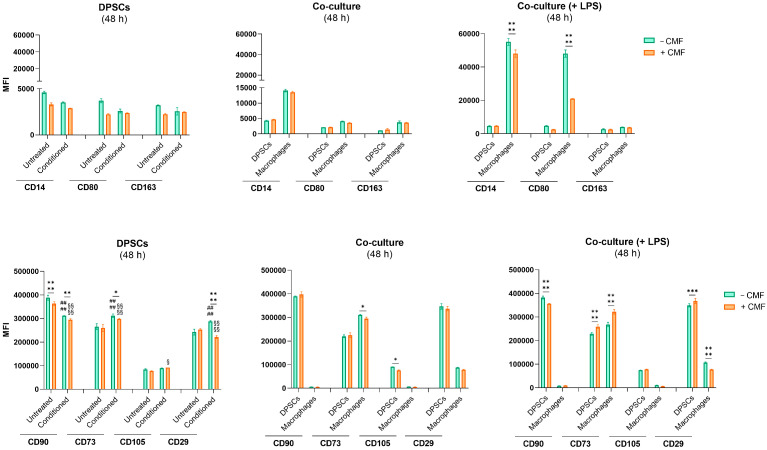
Immunophenotypic profile of cells exposed to the CMF treatment after 48 h. The positivity of cells for CD14-FITC, CD80-PE, CD163-PE, CD90-FITC, CD73-PE, CD105-PE, and CD29-PE is proportional to the MFI (mean fluorescence intensity). * *p* < 0.05, ** *p* < 0.01, *** *p* < 0.001. and **** *p* < 0.0001 between cells exposed to the CMF treatment and cells not exposed; #### *p* < 0.0001 between conditioned DPSCs and untreated DPSCs without CMF; § *p* < 0.01 and §§§§ *p* < 0.0001 between conditioned DPSCs and untreated DPSCs with CMF.

A co-culture system was established, as previously described, and visualized by light microscopy after crystal violet staining (Figure 5a). The characteristic spindle-shaped morphology of DPSCs is clearly observable. DPSCs and LPS-stimulated macrophages were differentiated by flow cytometry based on CD14 expression (Figure 5b). DPSCs exhibit weak CD14 staining, whereas macrophages, activated by LPS, strongly express this marker. Additionally, when examining the fluorescence emission related to CD14 on the morphological dot plot (SSC-A/FSC-A), the light scatter patterns of both cell types align with their respective sizes and complexities. DPSCs (in blue) are spread across the high FSC-A and medium SSC-A regions, indicating larger size and moderate complexity. In contrast, macrophages (in red) are mainly located in the lower FSC-A region, reflecting their smaller size. CD14, CD80, and CD163 expression levels are notably higher in macrophages compared to DPSCs (Figure 5c and Figure 6). CD80, a key marker associated with M1 macrophage polarization, exhibits an exponential increase following LPS exposure in the co-culture system after 48 h. Treatment with CMF leads to a significant reduction in CD80 expression, cutting its levels by approximately 50%. CD14, an LPS-binding protein expressed in monocytes and most tissue macrophages, exhibits a pronounced upregulation in response to LPS exposure, similar to CD80. However, CD14 expression is modestly reduced only 48 h after treatment with CMF. CD163, a marker of M2 macrophages, does not exhibit significant expression modification upon LPS exposure. Furthermore, CMF does not induce a notable modulation of CD163 expression. For the identification of Mesenchymal Stem Cells (MSCs), a minimal phenotypic pattern requires the cells to express CD90, CD73, CD105, and CD29 (Figure 5c and Figure 6). No significant differences in CD90 expression are observed in untreated and conditioned DPSCs at 24 and 48 h. However, CD90 expression decreases when DPSCs are exposed to conditioned medium, with CMF treatment inducing a slight additional reduction in both untreated and conditioned cells. Similarly, CD90 levels remain elevated in DPSCs co-cultured with macrophages, but CMF treatment reduces its expression following LPS exposure at the 48-h time point. Within the first 24 h, CD73 expression is upregulated in both untreated and conditioned DPSCs when exposed to CMF. A similar trend is observed in the co-culture systems, regardless of LPS presence at 24 h and in LPS-stimulated-co-cultures at 48 h. Macrophages also express CD73, but no modulation is detected within 24 h. At 48 h, LPS-stimulated macrophages co-cultured with DPSCs show a significant increase in CD73 expression upon CMF treatment. CD105 expression in DPSCs remains unchanged in all the experimental conditions. In contrast, CD105 is highly expressed in macrophages co-cultured with DPSCs within the first 24 h, though this expression is not observed at 48 h. Exposure to conditioned medium enhances CD29 expression in DPSCs. However, CMF treatment downregulates CD29 in conditioned DPSCs and upregulates it in untreated DPSCs. Both DPSCs and macrophages express CD29, with CD29 levels significantly increasing in DPSCs after 48 h. CMF half-reduces CD29 expression in DPSCs within the first 24 h, regardless of LPS exposure. After 48 h, DPSCs co-cultured with macrophages show a slight increase in CD29 expression, whereas macrophages exhibit decreased CD29 expression.

## 3. Discussion

Dental caries is a prevalent condition that, in extreme circumstances, results in pulp necrosis in addition to destroying the teeth’s hard structure. The most typical therapy for pulp necrosis is to remove the damaged pulp tissue, which results in a decrease in tooth vitality and an increase in tooth fragility [1]. The mesenchymal stem cell-like properties of dental pulp stem cells (DPSCs), which are extracted from pulp tissue, make them excellent candidates for repairing injured dental pulp tissue [10]. The interaction between immune cells and stem cells is important during tissue repair. Characterizing the interplay between immune cells and stem cells is crucial to understanding how to improve natural repair mechanisms [11].

For this reason, in the present work, DPSCs were conditioned with the medium from LPS-stimulated macrophages, and a co-culture model of DPSCs and macrophages under basal and pro-inflammatory conditions has been established. Under these experimental conditions, we demonstrated that CMF treatment can restore the redox balance in terms of cytotoxicity occurrence and generation of superoxide anions. It has been well demonstrated that the maintenance of redox homeostasis is crucial for the vital function of DPSCs, such as the odontogenic commitment [12]. Notably, CMFs are found to modulate membrane CD markers related to macrophage polarization and the odontogenic commitment of DPSCs [8,9].

Dental pulp is a nerve- and vascular-rich mesenchymal connective tissue surrounded by a mineralized tissue called dentin. When the original odontoblasts are lost due to severe pulp damage, the pulp tissue, which contains DPSCs, mesenchymal stem cells with fibroblastic characteristics that can differentiate into a new generation of odontoblast-like cells, is responsible for reparative dentin formation [13].

In a clinical situation, after injuries or bacterial infiltrations, cytokines are released from odontoblasts and probably mesenchymal pulp cells to finally initiate dental pulp inflammation as a mechanism of defense. These adaptive cell responses are related to oxidative and nitrosative stress, caused by Reactive Oxygen or Nitrogen Species (ROS or RNS, respectively). Dysregulated high levels of ROS or RNS may lead to apoptosis and cell cycle arrest in MSCs [14]. In the first step of the oxidative metabolism, NADPH oxidase (NOX), with the help of its cytosolic subunits p67, 47, and 40, and membrane proteins like p22phox, converts NADPH to NADP+ and generates superoxide anions [15]. In our experimental model, increased generation of superoxide anions is paralleled by the augmented production of LDH under pro-inflammatory conditions (conditioned DPSCs and co-culture + LPS), suggesting oxidative stress-induced cytotoxicity. In the same experimental conditions, CMF treatment can counteract oxidative stress after 24 h, the amount of superoxide anions generated considerably decreased as the release of LDH (Figure 3 and Figure 4). Since it has been reported that the relevance of cellular redox homeostasis for vital functions of human dental pulp cells [12], the modulation of markers expressed on cell membranes of both DPSCs, and macrophages has been analyzed.

DPSCs express phenotypic markers including CD29, CD90, CD105, and CD73, suggested to characterize cells as stem cells in vitro, while they are negative for CD45 and CD14, markers typically expressed by hemopoietic and immune compartments [16]. Different types of immunocompetent cells are found within the dental pulp tissue, including resident macrophages. These macrophages can be polarized into classically activated macrophages (M1 type), which serve an inflammatory role, and alternatively activated macrophages (M2 type), which fulfill an anti-inflammatory function. The proportion of M1 or M2 macrophages or the temporal sequence of their appearance is essential for tissue remodeling [17,18]. CD163 is a macrophage specific scavenger receptor for haptoglobin-hemoglobin complexes found on the cell membranes of M2 macrophages. Its expression is strongly induced by the anti-inflammatory cytokine IL-10, making CD163 a marker of the anti-inflammatory process occurrence [19]. On the contrary, CD80^+^ cells are classically M1 macrophages [20]. In parallel, CD14 is a glycolipid-anchored membrane glycoprotein expressed on cells of the myelomonocyte lineage, including monocytes, macrophages, and some granulocytes. CD14 is a key molecule in the activation of innate immune cells and is an essential part of the LPS receptor complex [21]. In our experimental model, both under basal and pro-inflammatory conditions, DPSCs are negative for macrophage-related markers (CD14, CD80, and CD163), as expected. On the other hand, CD14 is significantly upregulated in macrophages in the co-culture system stimulated by LPS (Figure 5 and Figure 6). After 24 h from the CMF treatment, a downregulation of CD14 is registered in the co-culture system stimulated by LPS, and this effect is even amplified after a second cycle of treatment (48 h), as a sign of escape from the LPS-induced inflammation.

The positivity of MSCs from the dental pulp to the various cell markers is differentially modulated under the odontogenic commitment [22]. CD90 (Thy-1) is classically decreased in a time-dependent manner, meaning that cell stemness is reduced towards osteogenic differentiation [23], whereas CD73 is upregulated under osteogenic conditions and highly expressed in odontoblasts, as it is involved in adenosine production and in the enhancement of bone metabolism through the Wnt-β-catenin signaling [24]. In our experimental model, markers are not modulated after 24 h from treatment in DPSCs, while a slight but significant modulation of CD90 and CD73 can be detected after a second cycle of CMFs, in alignment with literature (Figure 6). It is also reported that CD73-triggered signal stimulates a shift from an ATP-driven pro-inflammatory environment to an anti-inflammatory niche induced by adenosine in macrophages [24]. In the co-culture model + LPS, there is a slight but significant increase in CD73 expression, making it plausible to assume that macrophages are trying to shift their metabolism to favor anti-inflammatory responses.

## 4. Materials and Methods

### 4.1. Cell Cultures

Human dental pulp stem cells (DPSCs-PT-5025) and undifferentiated human monocytes (CRL-9855^TM^) were purchased from Lonza (Lonza Group Ltd., Basel, Switzerland) and ATCC^®^, respectively. DPSCs were grown in α-MEM (Gibco, ThermoFisher Scientific, Waltham, MA, USA) and CRL-9855 ^TM^ were cultured in RPMI 1640 (Merck, Darmstadt, Germany) at 37 °C and 5% CO_2_. Both media were supplemented with 10% heat-inactivated fetal bovine serum (FBS, Gibco, ThermoFisher Scientific, Waltham, MA, USA) and 1% penicillin/streptomycin (EuroClone S.p.a., Milan, Italy), whereas 1% sodium pyruvate (Merck, Darmstadt, Germany) was added only to RPMI 1640.

Monocytes were differentiated by stimulation with 100 ng/mL of PMA (phorbol-12-myristate-13-acetate, purchased from Merck, Darmstadt, Germany, stock solution 1 mM in DMSO) in complete RPMI for 48 h at 37 °C and 5% CO_2_.

### 4.2. Set up of a Cell Co-Culture Model and Establishment of Pro-Inflammatory Conditions

To set up the co-culture model, differentiated macrophages were harvested with StemPro™ Accutase™ (Merck, Darmstadt, Germany), collected by centrifugation, and seeded together with DPSCs in a 24-well plate at a ratio of 5 (macrophages) to 1 (DPSC) in RPMI 1640 (2.4 × 10^4^ macrophages and 0.7 × 10^4^ DPSCs). LPS (lipopolysaccharide from *E. coli* purchased from Merck, Darmstadt, Germany, stock solution 1 mg/mL in water) was added to the cultures at a final concentration of 0.5 µg/mL to establish an inflamed environment. The cells were then incubated for 24 h. Following this incubation period, the cells were exposed to the treatment. In parallel, DPSCs were either treated under basal conditions or conditioned with the medium collected from LPS-stimulated macrophage cultures for 24 h and afterwards exposed to the treatment. Therefore, the experimental conditions were the following: untreated DPSCs (DPSCs under basal conditions), conditioned DPSCs (DPSCs stimulated with the medium from LPS-stimulated macrophages for 24 h), co-culture (DPSCs/macrophages 1:5), and co-culture + LPS (DPSCs/macrophages 1:5 with LPS).

### 4.3. Cell Treatment with the CMF Device

**Figure 7:**
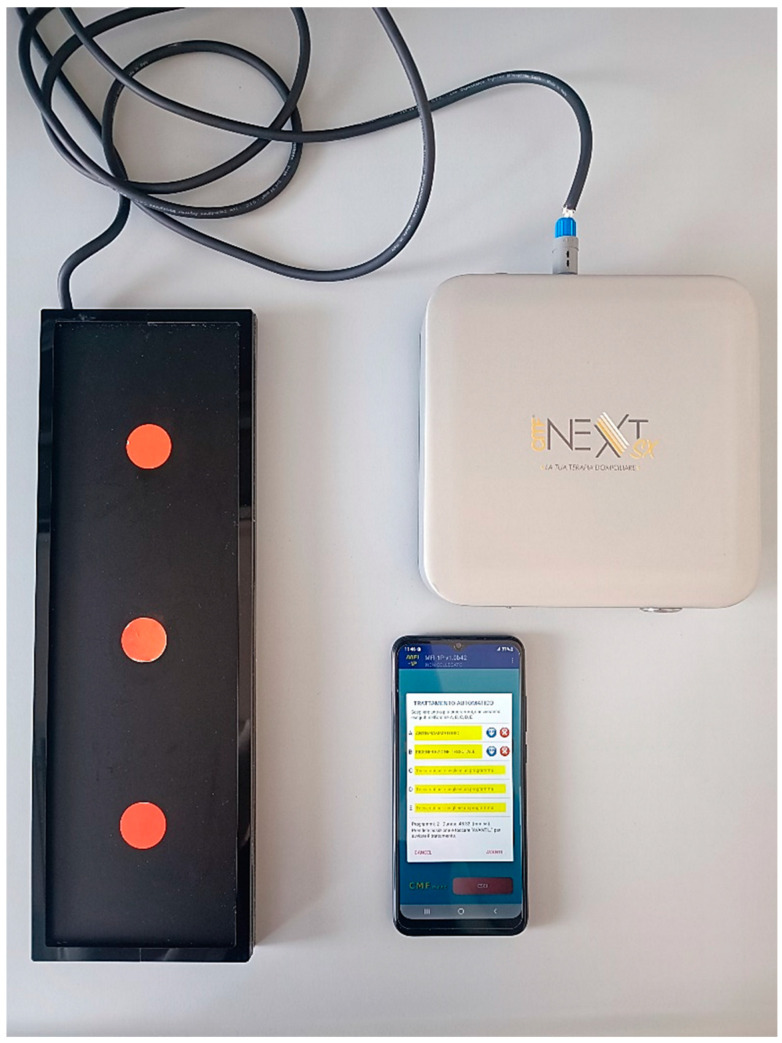
CMFs instrument, Next SX version (M.F.I. Medicina Fisica Integrata, Rome, Italy) equipped with a CMF generator, a support for plates and flasks, and a smartphone supporting the MFI-1P software (version 5.0, M.F.I. Medicina Fisica Integrata, Rome, Italy) programming the CMF generator.

The CMF instrument, Next SX version (M.F.I. Medicina Fisica Integrata, Rome, Italy), is an electronic device that emits innovative pulsed multi-frequency electromagnetic fields (Figure 7). These fields range in intensity from 1 to 250 microTeslas (µT) and can be varied in terms of intensity, frequency, waveform, and timing of stimulation [8]. The CMF generator is equipped with various programs that operate in relation to the configuration of the specific sector of the application. Each program is composed of several steps set at different intensities (1–250 µT), frequencies (1–250 Hz), interval times (1–4 min for each step), and forms of the complex multi-frequency waves with harmonic enrichments. Those four parameters represent one of the steps of the machine program. Additional details regarding machine programs are patent pending. Two distinct programs were used: Program A (anti-inflammatory) and Program B (tissue regeneration), with a total duration of 48 min and 32 s. Cells cultured in 24- or 6-well plates were properly positioned on the device, and the treatment was carried out inside the incubator at 37 °C with 5% CO_2_. The treatment was performed twice, every 24 h. Samples were harvested and labeled at 24 h and 48 h.

Images of cells were acquired 24 and 48 h after treatment by means of a light phase-contrast microscope equipped with a camera (Leica). Images were acquired and analyzed by the Leica Application Suite LAS EZ version 3.4 (Leica, Wetzlar, Germany).

### 4.4. Cytotoxicity Assay (LDH Test)

The CytoTox 96^®^ Non-Radioactive Assay (Promega Corporation, Fitchburg, WI, USA) was used to quantify Lactate Dehydrogenase (LDH) released in cell supernatants 24 and 48 h after treatment. This assay measures LDH, a stable cytosolic enzyme released upon cell lysis. Released LDH in culture supernatants is measured with a 30-min coupled enzymatic assay, which results in the conversion of a tetrazolium salt (iodonitrotetrazolium violet; INT) into a red formazan product. The absorbance signal was measured at 490 nm with a spectrophotometer (Multiskan GO, Thermo Scientific, Monza, Italy). The results were expressed according to the formula: LDH released (fold increase on the control) = [(A − B)/(C − B)], with A = LDH activity of sample, B = LDH activity of growth medium (background) and C = LDH activity of the untreated control [25].

### 4.5. Detection of Mitochondrial Superoxide Anions

Cells were cultured in a 6-well plate (1.5 × 10⁵ cells per well) using complete routine culture medium and treated as previously described. The formation of Reactive Oxygen Species (specifically Mitochondrial Superoxide Anion) was detected by flow cytometry. Cells were stained with the fluorescent dye MitoSOX Red (Thermo Fisher, Waltham, MA, USA) at a concentration of 1 µL/mL (stock solution 1mM in DMSO), and the relative fluorescence emission was measured using a Beckman Coulter CytoFLEX flow cytometer (Brea, CA, USA). Data were analyzed with the CytExpert Software 5.0 version (Beckman Coulter, Brea, CA, USA), and results were expressed as mean fluorescence intensities (MFIs).

### 4.6. Immunophenotyping In Vitro by Flow Cytometry

The expression of surface markers (CDs) was analyzed by flow cytometry. Cells were cultured in a 6-well plate using complete routine culture medium, and after treatments, they were harvested, collected by centrifugation in the cold, and washed once with FACS buffer. Cells were incubated with fluorochrome-conjugated antibodies (1:50 dilutions) in 50 μL of FACS buffer for 15 min in the dark. Cells were incubated separately in each single screening tube with anti-human monoclonal antibodies, CD14-FITC, CD80-PE, CD163-PE, CD90-FITC, CD73-PE, CD29-PE, and CD105-PE (BD Biosciences, Franklin Lakes, NJ, USA). Then, the excess of antibodies was removed by adding fresh FACS buffer and by centrifugation. 10,000 events were run in a Beckman Coulter CytoFLEX flow cytometer (Brea, CA, USA). Relative fluorescence emissions of gated cells by Forward and Side Scatter properties (FSC/SSC) were analyzed using the CytExpert Software 5.0 version (Brea, CA, Beckman Coulter), and results were expressed as the percentage of positive cells for each CD marker [26].

### 4.7. Statistical Analysis

Statistics were performed using one-way analysis of variance (ANOVA) followed by Dunnet and Tukey’s multiple comparison tests by means of the Prism 8.0 software (GraphPad, San Diego, CA, USA). Results are presented as mean values ± standard deviations. Values of *p* ≤ 0.05 were considered statistically significant.

## 5. Conclusions

In the present work it has been demonstrated that CMFs are able to enhance DPSC proliferation under pro-inflammatory conditions. Moreover, LPS-induced cytotoxicity as well as superoxide production are decreased by the CMF treatment. The modulation of CD90, CD105, CD73, and CD29, crucial markers in the odontogenic commitment of DPSCs, in parallel with CD14, which is involved in the M1 polarization of macrophages, is positively modulated at 48 h after a double treatment with CMFs. All in all, data suggest that Complex Magnetic Fields, by modulating the odontogenic commitment and the anti-inflammatory response of dental pulp stem cells, can represent a good therapeutic tool against pulpitis and peri-implantitis.

## Data Availability

Data are contained within the article.
